# GP turnover in a multiprofessional team-based primary care system: evidence from Sweden

**DOI:** 10.1080/02813432.2025.2587544

**Published:** 2025-11-19

**Authors:** Lina Maria Ellegård, Anders Anell, Gustav Kjellsson

**Affiliations:** a Department of Economics, Lund University School of Economics and Management, Sweden; b Department of Economics and Business Law, Kristianstad University, Sweden; c Department of Business Administration, Lund University School of Economics and Management, Sweden; d School of Public Health and Community Medicine, Sahlgrenska Academy, University of Gothenburg, Sweden; e Centre for Health Governance, Department of Economics, University of Gothenburg, Sweden

**Keywords:** Practice turnover, continuity of care, organisation, retention, general practice

## Abstract

**Background:**

GP recruitment and retention difficulties challenge the traditional general practice model. Task-shifting and relieving GPs from financial risk have been suggested to make primary care more attractive. In Sweden’s multiprofessional team-based primary care system, GPs usually work as salaried employees and there is extensive task-shifting. Salaried employment facilitates mobility, potentially leading to high turnover. The opportunity to work on fixed contracts can also increase turnover rates.

**Aim:**

To describe practice turnover rates and examine associations with practice characteristics in a Swedish region.

**Design and Setting:**

Analysis of observational register data from Skåne, Sweden (1.4 million residents).

**Method:**

Turnover rates were calculated for 157 primary care practices in 2010–2018. The main dataset included all physicians – permanent and temporary workers – regularly providing care in each month. To understand the role of temporary workers, a supplementary analysis was performed on permanently employed GPs and registrars at 80 public practices in 2019. Associations between turnover and practice characteristics were examined in bivariate analyses and multiple regressions.

**Results:**

Annual practice turnover rates ranged between 20–40% (mean 30%), showing no time trend. The high rates mainly reflected the use of temporary GPs; in the supplementary analysis of permanent GPs and registrars, the mean annual turnover rate in 2019 was 13-15%. Turnover was higher for practices with socially deprived patients or high workload. Private practices had lower turnover conditional on the higher workload.

**Conclusion:**

The results indicate that a primary care system with salaried GPs facilitates GP mobility, which in turn creates barriers to continuity of care.

## Introduction

Many countries struggle with GP recruitment and retention difficulties [1]. An adequately sized and stable workforce is key to ensure access and continuity of care – fundamental aspects of primary care [2] – and enables local quality improvement work [3].

Policy proposals to increase the attractiveness of primary care include task-shifting to other professionals [4,5] and relieving GPs from financial risk [1,6]. In Sweden, such features are institutionalised in the traditions of a multiprofessional team-based primary care system [7]. There is extensive task-shifting, with nurses performing triage, preventive activities and treatment of minor ailments, and follow-up of chronic conditions. GPs are salaried employees, thus protected from the financial and administrative hardships associated with running a practice [8]. Yet, the GP density is one third lower than the EU average [9]. Relational continuity of care, in terms of frequency of visits with a regular GP, is low, indicating high turnover [10]. An intriguing possibility is that the features that have been suggested to relieve GP shortage also induce high turnover: Task-shifting challenges GPs’ professional role as generalists, possibly reducing work satisfaction. Salaried GPs can quit with short notice and minimal paperwork, and GPs who value flexibility can contract with practices on a short-term basis as freelance workers. In combination, these factors may increase turnover.

Although the institutional features make Sweden an interesting case, the GP turnover has not been systematically documented before. This study aims to explore practice turnover rates and to examine associations with practice characteristics in a Swedish region.

## Methods

### Setting and data

Sweden’s health care system is operated by 21 independent regions. Developments in primary care since the early 1970s have favoured a multiprofessional team-based approach, although geographical responsibility was replaced by regional markets with individual choice of eligible private or public practices in 2010 [11]. Each region individually regulates the requirements that primary care centres (PCCs) must meet to receive public funding. There is no restriction on entry for new PCCs that meet these requirements.

Our study setting, Region Skåne, has 1,4 million residents and approximately 160 PCCs (average number of patients/PCC is 8,665, Table 1). 55% of the PCCs are publicly owned. 39 of the 70 private PCCs in our analysis belonged to three nationally operating investor-owned chains. Of the others, nine were owned by a national GP partnership (*Praktikertjänst*), seven by local chains (typically physician-owned) with 2–3 PCCs, and 15 were independent PCCs owned by physicians (of which only one was a solo practice).

**Table 1.** Patient and PCC characteristics.

Characteristic	Definition of original variable and summary statistics	Definition in main analysis
Size	Registered patients by PCC and month (continuous). Mean (std.dev) = 8,665 (3,802)	0 = missing 1 = lowest tercile 2 = mid tercile 3 = highest tercile
Morbidity	Casemix (ACG) relative to the regional mean (continuous). Mean (std.dev) = 1.01 (0.11)	0 = missing 1 = lowest tercile 2 = mid tercile 3 = highest tercile (highest mobidity)
Social deprivation	CNI (Care Need Index) relative to the regional mean (continuous). Mean (std.dev) = 1.02 (0.36)	0 = missing 1 = lowest tercile 2 = mid tercile 3 = highest tercile (most deprived)
Ownership	Type of owner of the PCC (public or private) Share public: 55%	0 = public 1 = private
Location	Municipality where the PCC is located. Distribution: City 49%, Commuting 11%, Town 17%, Rural 22%	0 = City 1 = Commuting area 2 = Town 3 = Rural
Workload	Registered patients per GP in workforce Mean (std.dev.): 1,502 (427)	1 = lowest tercile 2 = mid tercile 3 = highest tercile (highest workload)

PCCs are staffed by physicians and nurses, but also other care professionals (e.g. physiotherapists and psychotherapists). Most GPs in both public and private PCCs are salaried employees rather than practice-owners, even though both employment and ownership arrangements may differ across different types of private PCCs. For instance, all GPs are salaried employees in both the national chains and the national GP partnership, but in the latter case some of the GPs are also shareholders in the national organization and responsible for the practice operations.

For unfilled vacancies (due to recruitment problems or temporary leaves), a PCC can contract with freelance GPs (directly or *via* a staffing agency) or hire GPs on fixed contracts. Trainees – foundation doctors and registrars – also provide consultations. Foundation doctors are employed by the region for 18 months, around six of which are spent at PCCs. Registrars specialising in general medicine are employed at a PCC for around 5 years but spend part of the time outside the primary care setting.

To measure turnover, we used the regional care database covering all consultations provided by physicians (including freelancers and trainees) in public and private PCCs. Data on PCC characteristics was sourced from the region and prior research.

The care database does not distinguish between contract types; e.g. we cannot observe if the physician held a permanent position or not. Temporary workers (freelancers, fixed contracts and trainees) by definition have high turnover. To understand how temporary workers contribute to the overall turnover rate, we obtained supplementary data on GPs and registrars on permanent contracts from the region’s human resource department. This data was limited to public PCCs and only available from 2019. See Table S1 for a comparison of the two data sources.

### Study population

For the main analysis (turnover for all contract types), the study population included 157 PCCs operating for ≥2 years in 2010–2018. There were 1,304 PCC-year observations in the panel, which was unbalanced due to openings and closures. For the 11 PCCs that closed, we excluded the closure year. We examined a balanced panel (113 PCCs) in a sensitivity analysis. Setting 2018 as the final year allowed us to study the annual turnover through 2018. We also considered the turnover within horizons of up to five years, using 2014 as the last year.

The supplementary analysis of turnover among permanently employed physicians covered 80 public PCCs in 2019–2024.

### Variable definitions

#### Workforce and GP-PCC spells

We defined the workforce of a PCC in a given month as consisting of all physicians providing consultations on at least 10 days (mean = 5.9 GPs, IQR 3.6–7.9). Other definitions (15/20 days; mean = 3.7/1.1 GPs) were used in a sensitivity analysis.

A period during which a GP regularly provided care at a PCC was denoted a *spell.* A spell was defined to end after a break between consultations of at least 365 days. This definition allowed for leaves of up to one year. The spell duration was calculated as the number of days between the first and the last consultation. Figure S1 illustrates an example of a GP with two spells in one PCC.

#### Turnover rate by PCC and year

For the main analysis, we calculated the annual turnover rate for each PCC and month *m* as

(1) Turnoverjm=Physicians  in  workforce  whose  spell ended within  12  monthsPhysicians  in  workforce  of  PCC  j  month  m×100

We also calculated turnover within 6, 18, 24, 36, 48 and 60 months.

As the PCC-month level measure changes slowly, we aggregated at the PCC-year level by averaging over all months within each year and PCC:

(2) $${\text{Turnover}}_{\text{jy}}=(\sum_{m=1}^{12}{\text{Turnover}}_{\text{jm}})/12\;$$

For 25 PCC-year observations with no regular workforce (15 recently established PCCs), we imputed the mean value of each PCC.

For the supplementary analysis of turnover among permanent GPs in public PCCs, we could only calculate the annual turnover relative to the January workforce:

(3) $$\text{Turnove}{\text{rPermanentGP}}_{\text{jy}}=\frac{\text{Permanently employed phys}i\text{cians quitting during year}}{\text{Permanently employed physicians in January}}\times 100$$

#### Turnover rate by PCC

When examining associations with PCC characteristics, we used a time-invariant (PCC-level) measure, i.e. the average of annual turnover rates ($Y_{i}$=number of years):

(4) $$\bar{{\text{Turnover}}_{j}}=(\sum_{y=1}^{Y_{i}}{\text{Turnover}}_{\text{jy}})/Y_{i}\;$$

#### PCC characteristics

Table 1 describes PCC characteristics. Morbidity was measured by a risk score from an age-gender-diagnosis-specific prediction model (Adjusted Clinical Groups^®^). Social deprivation was measured by an index capturing registered patients’ characteristics (educational level, foreign background, unemployment, single residency among elderly, single parenthood, age, and within-country migration) which is highly correlated with low income [12]. The workload was calculated by dividing the number of registered patients by the workforce.

We categorized continuous measures by classifying PCCs into terciles of the distribution of PCC-level averages.

### Methods

We calculated summary statistics of PCC turnover rates and illustrated the data using boxplots.

Relationships between the average annual turnover (${\text{Turnover}}_{12j}$) and PCC characteristics were explored in bivariate analyses and multiple regression models with heteroskedasticity-robust standard errors. The full regression specification was:

$${\text{Turnover}}_{12j}=\alpha +\beta_{1}\;{\text{Size}}_{12j}+\beta_{2}\text{Morbidity}+\beta_{3}\text{Deprivation}+\beta_{4}\text{Private}+\beta_{5}\text{Location}+\beta_{5}\text{Workload}+\varepsilon_{j}$$

where α is a constant and $\varepsilon_{j}$ an error term. Analyses were performed in Stata 18.1.

## Results

### Annual turnover

**Figure 1.**
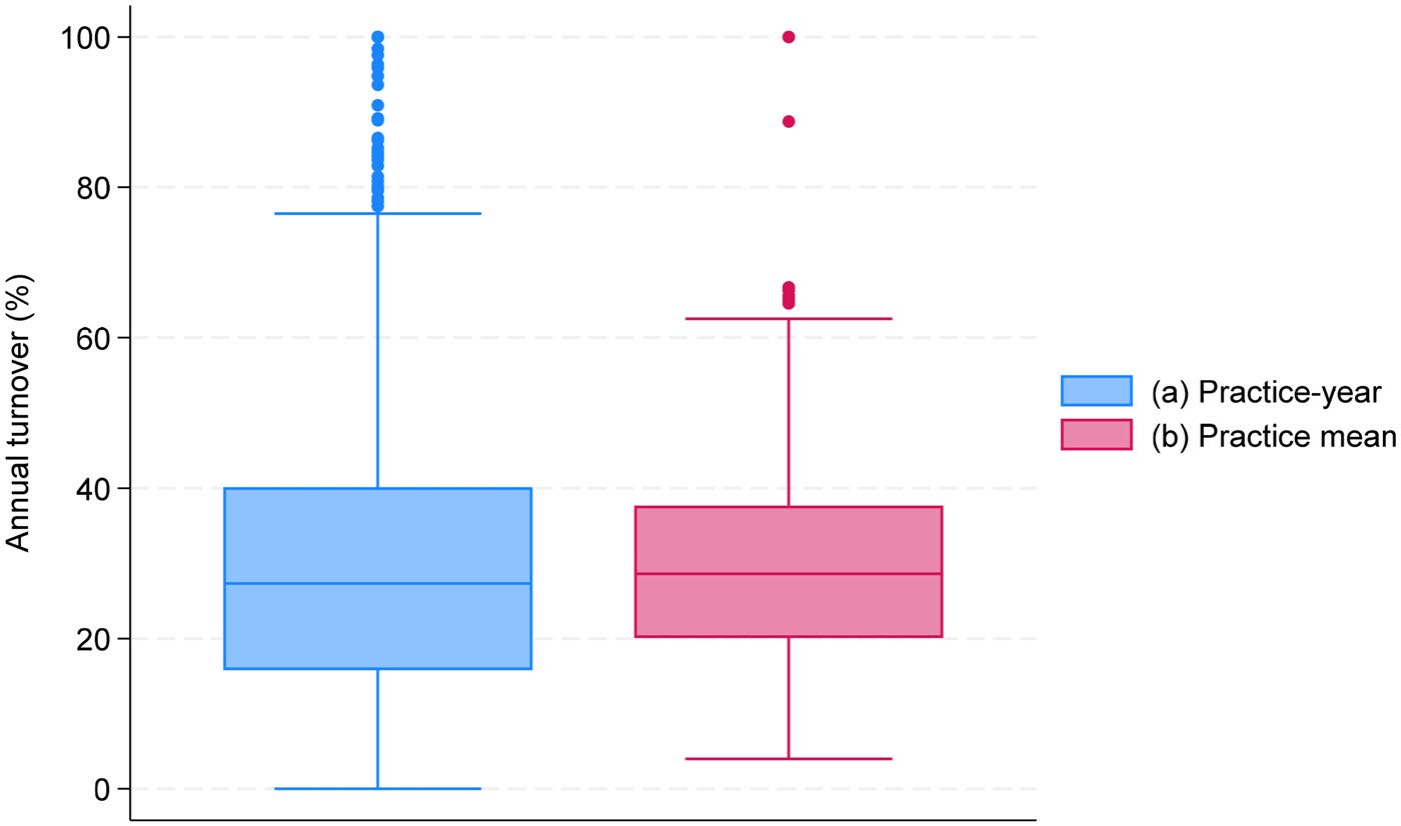
Annual turnover rate. (a): PCC-year level (${\text{Turnover}}_{\text{jy}}$). (b): PCC-level ($\bar{{\text{Turnover}}_{j}}$). *Note:* Each box indicates the median and inter-quartile range (p75-p25), whiskers show the upper and lower adjacent values (p75 (p25) + (-) 1.5*(p75-p25)), dots represent observations outside this range.

Figure 1 illustrates the annual turnover rates of the total workforce (all contract types) at the PCC-year (1a) and PCC (1b) level. For both measures, the median and the mean (not in figure) rates were 30% and the inter-quartile range was around 20-40%.

The distributions did not show a time trend (Figure A1) and were similar for the balanced sample (Figure A2). The medians were similar for other definitions of the regular workforce; the IQR was stable for the 15-day definition but wider with the 20-day definition (Figure A3).

**Figure 2.**
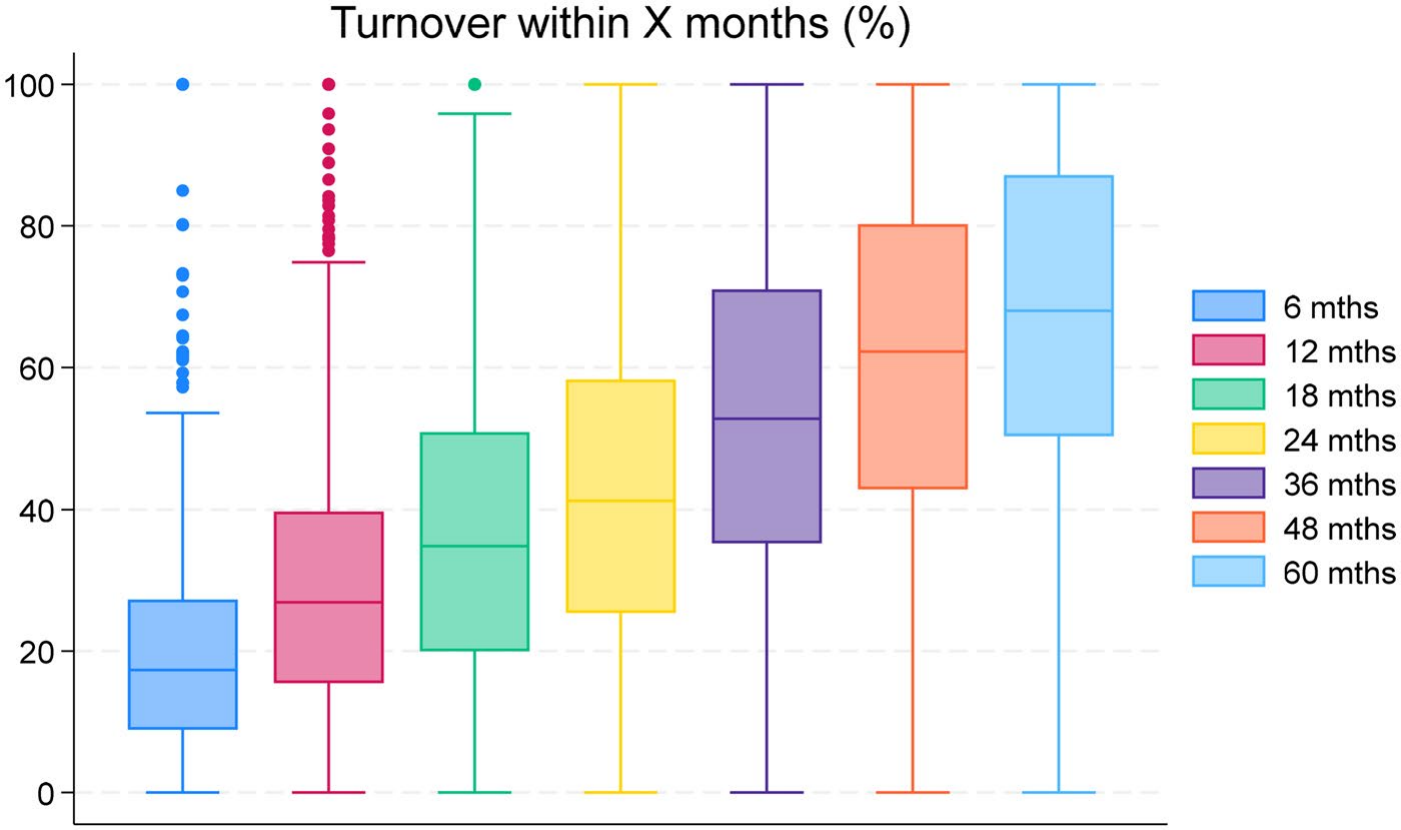
Turnover by length of follow-up. PCC-year-level data for 2010–2014. *Note*: Each boxplot shows the distribution within a specific horizon. Leftmost: turnover within 6 months etc.

**Figure 3.**
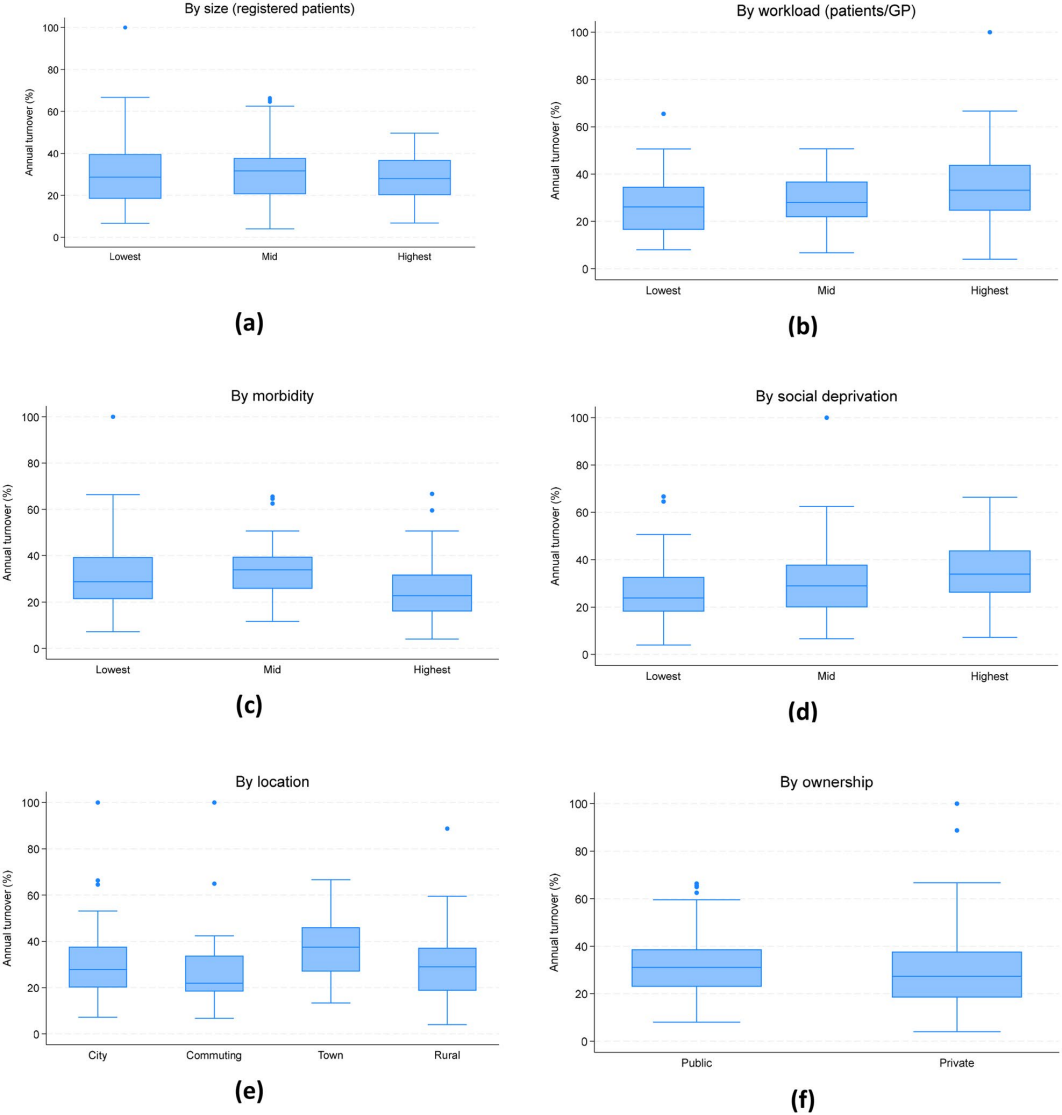
Bivariate analysis.

Figure 2, which considers other time horizons, shows that for the median PCC, one in five ongoing spells end within six months. Within five years, 67% of spells have ended.

### Permanent vs temporary workers

In the dataset on permanently employed GPs, the mean turnover rate in 2019 (the year closest to the care production data) was 15% for GPs and 13% for registrars (in 2020–2024, the rates were 12-19% and 9-13%). The annual turnover rate of 30% for the whole workforce (including temporary workers) thus to a large extent reflects the - by definition – high turnover of doctors on short-term contracts. Combining the rates, we estimate that approximately one-fifth of the regular workforce in a given month are freelancers, temporarily employees or foundation doctors.^1^

### Associations between annual turnover and PCC characteristics

According to the bivariate analysis, turnover was unrelated to PCC size (Figure 3(a)) but increasing with the workload (Figure 3(b)). The relationship with morbidity had an inverse U-shape (Figure 3(c)). PCCs with more deprived patients had higher turnover (Figure 3(d)). There were no clear urban-rural differences; turnover was the highest in mid-sized towns (Figure 3(e)). The distributions for public and PCCs were similar, albeit with slightly lower rates in private PCCs (Figure 3(f)).

Most bivariate patterns persisted after adjusting for other characteristics (Table 2). However, a relationship with size emerged (the largest PCCs had 9% points lower turnover than the smallest, *p* < 0.01). Additionally, turnover was significantly lower in private PCCs (−6% points, *p* < 0.01) once adjusting for the higher workload in private PCCs. This difference was primarily driven by private PCCs owned by the national GP partnership, local chains or GPs; PCCs belonging to the national investor-owned chains were more similar to public PCCs in terms of turnover rates (Table A1). Notably, the turnover was 8-9% points higher in the most deprived tercile compared to the least deprived. The sensitivity analysis on the balanced sample (Table A2) gave similar results except that there was no association between morbidity and turnover.

**Table 2.** Regression models of average annual turnover.

	(1) Full	(2) Excl. private	(3) Excl. workload
Medium size	−2.606	−0.928	−0.949
	(3.030)	(3.167)	(3.059)
Largest size	−9.190**	−6.569*	−7.065*
	(3.101)	(3.244)	(3.095)
Medium workload	5.060**	4.431*	
	(1.846)	(1.830)	
Highest workload	10.323***	7.682**	
	(3.042)	(2.876)	
Medium morbidity	1.883	1.451	1.145
	(2.485)	(2.483)	(2.532)
Highest morbidity	−8.793**	−10.543***	−10.753***
	(2.915)	(2.780)	(3.029)
Medium deprivation	2.967	3.548	3.263
	(2.672)	(2.637)	(2.789)
Most deprived	8.534**	8.702**	7.659**
	(2.629)	(2.673)	(2.711)
Commuting	−0.016	−0.504	−1.051
	(3.054)	(3.047)	(3.054)
Town	9.888**	10.527**	11.535***
	(3.087)	(3.142)	(3.012)
Rural	0.065	0.937	1.687
	(2.976)	(3.055)	(2.915)
Private	−5.931**		−2.363
	(2.099)		(2.220)
Constant	28.391***	25.568***	31.325***
	(3.506)	(3.776)	(3.687)
N	157	157	157

Note: Coefficients and robust standard errors in parentheses. * *p* < 0.05, ** *p* < 0.01, *** *p* < 0.001. The reference category is a public PCC which is located in a major city and belongs to the lowest tercile of the distributions of size, workload, morbidity and social deprivation. The dependent variable is the average of the annual turnover rates in 2010–2018.

## Discussion

### Summary

We calculated PCC turnover rates in Sweden based on all physicians providing care in a PCC and for permanently employed GPs in public PCCs. The mean annual turnover rate was 30% (IQR 20-40%) mainly due to the use of temporary staff. For permanent GPs in public PCCs the turnover was closer to 15%. Yet, the finding that two-thirds of all GPs leave the PCC within five years indicates high mobility also among permanently employed GPs.

Turnover was higher in areas with higher social deprivation. PCCs in towns had higher turnover than those in large cities, commuting and rural areas (which had similar rates). Private PCCs had lower turnover than public conditional on the workload, which by itself was positively associated with the turnover rate.

### Strength and limitations

We exploited a unique opportunity to explore turnover of all physicians providing care in a PCC, not just the permanently employed GPs, using rich administrative data from a setting with employed GPs.

A notable data limitation was the inability to distinguish between contract types. This was partly overcome by using supplementary data, which was however only available for public PCCs and starting one year after our study period. By implication, we could not examine if the difference between private and public PCCs reflect differences in the reliance on GPs on fixed contracts, differences in the turnover of permanently employed GPs, or both.

The care production data was only available through 2019. We believe that the results are still relevant, given the stability over time of turnover rates in both the main and supplementary analyses.

Our spell definition did not account for GPs returning to the same PCC and may therefore overestimate the long-run turnover. However, less than 10% of GPs ever returned to a PCC where they had worked previously.

We only counted heads and did not account for the share of work provided by each GP. Given the limited variation in the daily consultation volume across GPs (IQR: 6–8 visits/day), this is unlikely a severe limitation.

### Comparison with existing literature

The study suggests that high turnover is an important reason for the low relational continuity of care in terms of frequency of visits with a regular GP in Sweden [13,14]. The annual turnover rate was substantially lower (mean 10%) in a study of English practices [15]. One explanation is that they only studied contracted GPs. Another explanation is that most English GPs are practice-owners [16], thus facing higher exit barriers. This interpretation is supported by comparing our results to studies from Norway [17] and Denmark [18], with mainly self-employed GPs. While 68% of Norwegians are registered at the same GP for 4+ years, only 40% of residents in Skåne would be able to see the same GP four years later. 72% of elderly Danes has the same GP for 5+ years; that would only be feasible for 33% of residents in Skåne. In relation to this, the lower turnover rate we found for privately owned PCCs not belonging to a large investor-owned chain is notable. A potential explanation is that GPs in these settings have more vested interests in the PCC than physicians hired by PCCs owned by a national chain or the public sector.

Both the present study and [15] found higher turnover in more deprived areas. The lack of urban/rural difference in our study is surprising given the well-known GP shortage in rural areas [19]. One potential explanation is that salaried employment facilitates mobility in urban areas with many alternative PCCs to work with. Skåne is also relatively dense; the driving time to the closest city is around an hour even for the most rural PCCs.

### Implications for practice

The high turnover rates in Sweden underline the need to retain GPs and reduce the dependence on temporary workers, especially in deprived areas. The considerably lower turnover in countries with traditional GP-owned PCCs suggests that retention is higher when GPs have a vested interest in the PCC. Consistent with this interpretation, our main findings suggest that privately operated PCCs have lower turnover and our further analyses indicate that the difference is driven by PCCs owned by the national GP partnership, local chains or GPs, rather than by PCCs owned by national investor-owned chains. Further promoting a vested interest in the PCC among GPs in Sweden would likely require policies that facilitate physician ownership and/or foster new and innovative employment models that strengthen long-term commitment.

The mobility of Swedish GPs provides an important message for policymakers in other countries: organising primary care in a way that makes it less dependent on individual GPs may trigger a reciprocal response, with GPs starting to view PCCs as exchangeable, thus undermining the potential to build long-term patient-GP relationships. When reinventing general practice, policymakers need to prevent GPs’ attachment to their workplace from withering in the process.

## Supplementary Material

Turnover_Skane_Supplementary material_final_rev1_FINAL.docx

## Data Availability

The dataset used in the analysis is available on request.

## Credit statement

LME: Conceptualization; Data curation; Formal analysis; Funding acquisition; Investigation; Methodology; Software; Resources; Validation; Visualization; Writing – original draft; Writing – review & editing. AA: Conceptualization; Funding acquisition; Writing – review & editing. GK: Conceptualization; Data curation; Funding acquisition; Methodology; Resources; Writing – review & editing. MK: Conceptualization; Writing – review & editing.

## Note

1 Denote the proportion of permanent GPs (including registrars) by p. Setting their turnover to 14% (midpoint of 13% and 15%), the proportion of temporary workers (1-p) can be calculated by solving the equation 30% = 14%*p + 100%*(1-p).
