# Supplementary material for: GP turnover in a multiprofessional team-based primary care system: evidence from Sweden
Source: Scand J Prim Health Care. 2025 Nov 19;44(1):1–10. doi: 10.1080/02813432.2025.2587544 (PMC12918300; doi:10.1080/02813432.2025.2587544)
Supplement: Turnover_Skane_Supplementary material_final_rev1_FINAL.docx [file IPRI_A_2587544_SM2473.docx]

# Supplementary material

## Data Description

Table S1. Overview of datasets

| **Data Source** | **Period** | **Provider types** | **Basis for turnover calculation** | **Horizon for turnover calculation** |
| --- | --- | --- | --- | --- |
| Care production data  (Main analysis) | 2010-2019  Note that 2019 is used the follow-up period for the observations in 2018 (e.g., annual turnover relative to month *m* in 2018) | Public and private | All physicians in main workforce (defined by providing care at least 10 days in month *m*).  Includes permanently employed GPs, registrars, foundation doctors, GPs on fixed contracts, and freelance doctors. | Annual (main)  6 months – 60 months |
| Staff register  (Supplementary analysis) | 2019-2024 | Public | All permanently employed GPs and registrars in January. | Annual |

## Definition of location categories

City = Malmö, Lund, Helsingborg, Kristianstad.

Commuting = Staffanstorp, Burlöv, Vellinge, Kävlinge, Höganäs, Eslöv, Lomma, Svedala.

Town = Ystad, Trelleborg, Ängelholm, Landskrona, Hässleholm.

Rural= other (16 municipalities).

## Figures and tables

Fig S1 GP-practice spell definition

365 days

GP *i*,
practice *j*

Spell 1

Spell 2

*time*

*Note*: Green arrows = days when GP *i* provides consultations at practice *j*. Orange arrows = spell duration.

Figure A1 Boxplots by year

Figure A2 Boxplots for balanced panel. (a) Annual turnover (b) Average annual turnover

Figure A3: Boxplots for annual panel using different definition of the regular workforce

Figure A4 Boxplots for balanced panel at different lengths of the follow-up period.

Table A1 Distinguishing between private types

|  | (1) Main | (2) Private types | (3) Private types, excl. workload |
| --- | --- | --- | --- |
|  | b/se | b/se | b/se |
| Private | -5.931** |  |  |
|  | (2.099) |  |  |
| Private nat. chain |  | -4.849 | -0.736 |
|  |  | (2.476) | (2.277) |
| Private other |  | -7.322* | -4.741 |
|  |  | (3.526) | (3.804) |
| Medium size | -2.606 | -2.857 | -1.433 |
|  | (3.030) | (2.916) | (2.906) |
| Largest size | -9.190** | -9.403** | -7.529* |
|  | (3.101) | (2.989) | (2.952) |
| Medium workload | 5.060** | 4.777* |  |
|  | (1.846) | (1.900) |  |
| Highest workload | 10.323*** | 10.040** |  |
|  | (3.042) | (3.203) |  |
| Medium morbidity | 1.883 | 2.022 | 1.433 |
|  | (2.485) | (2.587) | (2.658) |
| Highest morbidity | -8.793** | -8.506** | -10.197** |
|  | (2.915) | (3.158) | (3.348) |
| Medium deprivation | 2.967 | 2.982 | 3.283 |
|  | (2.672) | (2.663) | (2.776) |
| Most deprived | 8.534** | 8.819*** | 8.177** |
|  | (2.629) | (2.592) | (2.649) |
| Commuting | -0.016 | 0.691 | 0.114 |
|  | (3.054) | (3.203) | (3.212) |
| Town | 9.888** | 9.747** | 11.231*** |
|  | (3.087) | (3.050) | (2.951) |
| Rural | 0.065 | -0.266 | 1.089 |
|  | (2.976) | (2.879) | (2.814) |
| Constant | 28.391*** | 28.542*** | 31.330*** |
|  | (3.506) | (3.476) | (3.739) |
| N | 157 | 157 | 157 |

Note: The leftmost column shows the main specification. Columns 2-3 distinguish between private providers that belong to a national investor-owned chain (Capio, Helsa, Prima Vård; n = 39) or has another owner (GP partnership or local chain with 2-3 PCCs; n = 31). Robust standard errors in parentheses. * p<0.05, ** p<0.01, *** p<0.001. The default category is a public practice which is located in a major city and belongs to the lowest third of the distributions of size, workload, morbidity and social deprivation.

Table A2 Regressions for the practices belonging to the balanced panel

|  | (1) Full | (2) Excl. private | (3) Excl. workload |
| --- | --- | --- | --- |
|  | b/se | b/se | b/se |
| Medium size | -0.336 | -0.138 | -0.872 |
|  | (2.851) | (3.044) | (2.972) |
| Largest size | -6.521* | -6.035 | -6.824* |
|  | (2.850) | (3.065) | (3.042) |
| Medium workload | 6.874** | 5.244* |  |
|  | (2.158) | (2.043) |  |
| Highest workload | 11.201** | 7.305* |  |
|  | (3.331) | (2.906) |  |
| Medium morbidity | 3.497 | 2.831 | 2.121 |
|  | (2.447) | (2.563) | (2.604) |
| Highest morbidity | -3.068 | -5.448 | -5.754 |
|  | (2.910) | (2.854) | (2.993) |
| Medium deprivation | 3.901 | 5.338* | 4.897 |
|  | (2.505) | (2.461) | (2.599) |
| Most deprived | 10.856*** | 13.067*** | 12.504*** |
|  | (2.666) | (2.741) | (2.953) |
| Commuting | -0.464 | 0.649 | 0.976 |
|  | (3.458) | (3.701) | (3.609) |
| Town | 5.988 | 8.348* | 9.802** |
|  | (3.363) | (3.365) | (3.449) |
| Rural | -1.326 | -0.628 | 0.516 |
|  | (2.924) | (3.033) | (3.045) |
| Private | -7.325** |  | -2.303 |
|  | (2.700) |  | (2.429) |
| Constant | 23.039*** | 21.800*** | 27.516*** |
|  | (3.328) | (3.526) | (3.211) |
| N | 113 | 113 | 113 |

Note: Robust standard errors in parentheses. * p<0.05, ** p<0.01, *** p<0.001. The default category is a public practice which is located in a major city and belongs to the lowest third of the distributions of size, workload, morbidity and social deprivation. Only practices existing all years. 113 practices open 2010-2018.
